# Hypoxia driven opioid targeted automated device for overdose rescue

**DOI:** 10.1038/s41598-021-04094-x

**Published:** 2021-12-31

**Authors:** Mohammad S. Imtiaz, Charles V. Bandoian, Thomas J. Santoro

**Affiliations:** 1 Department of Electrical and Computer Engineering, Bradley University, Peoria, IL 61612 USA; 2 Innovative Health Strategies, LLC, Peoria, IL 61614 USA

**Keywords:** Drug regulation, Biomedical engineering

## Abstract

Opioid use disorder has been designated a worsening epidemic with over 100,000 deaths due to opioid overdoses recorded in 2021 alone. Unintentional deaths due to opioid overdoses have continued to rise inexorably. While opioid overdose antidotes such as naloxone, and nalmefene are available, these must be administered within a critical time window to be effective. Unfortunately, opioid-overdoses may occur in the absence of antidote, or may be unwitnessed, and the rapid onset of cognitive impairment and unconsciousness, which frequently accompany an overdose may render self-administration of an antidote impossible. Thus, many lives are lost because: (1) an opioid overdose is not anticipated (i.e., monitored/detected), and (2) antidote is either not present, and/or not administered within the critical frame of effectiveness. Currently lacking is a non-invasive means of automatically detecting, reporting, and treating such overdoses. To address this problem, we have designed a wearable, on-demand system that comprises a safe, compact, non-invasive device which can monitor, and effectively deliver an antidote without human intervention, and report the opioid overdose event. A novel feature of our device is a needle-stow chamber that stores needles in a sterile state and inserts needles into tissue only when drug delivery is needed. The system uses a microcontroller which continuously monitors respiratory status as assessed by reflex pulse oximetry. When the oximeter detects the wearer’s percentage of hemoglobin saturated with oxygen to be less than or equal to 90%, which is an indication of impending respiratory failure in otherwise healthy individuals, the microcontroller initiates a sequence of events that simultaneously results in the subcutaneous administration of opioid antidote, nalmefene, and transmission of a GPS-trackable 911 alert. The device is compact (4 × 3 × 3 cm), adhesively attaches to the skin, and can be conveniently worn on the arm. Furthermore, this device permits a centralized remotely accessible system for effective institutional, large-scale intervention. Most importantly, this device has the potential for saving lives that are currently being lost to an alarmingly increasing epidemic.

## Introduction

Opioid use disorder has been designated as a worsening epidemic by the Center for Disease Control and Prevention (CDC). Approximately 100,000 people died from opioid-involved overdoses in 2021 according to the National Center for Health Statistics^1,2^, and the economic cost associated with such overdoses in the United States totaled $550 billion in 2017^3^. The increasing societal burden of the opioid use disorder and accompanying opioid-involved overdoses has raised the question of how deaths due to opioid overdoses can be better prevented.

While FDA-approved antidotes for opioid overdoses are available, such as naloxone^4^, and nalmefene^5^ they are effective in saving lives only if administered within a critically short time-frame after the overdose has occurred. Unfortunately, opioid-overdoses may occur in the absence of antidote^6^, or may be unwitnessed, and the rapid onset of cognitive impairment and unconsciousness, which frequently accompany an overdose may render self-administration of an antidote impossible^7^. Thus, many lives are lost because: (1) an opioid overdose is not anticipated (i.e., monitored/detected), and (2) antidote is either not present, and/or not administered within the critical window of effectiveness. The aim of this investigation is to design a safe, compact, non-invasive device that can monitor, and report an opioid overdose, and effectively deliver an antidote without human intervention.

Intravenous, and intraarterial, cannula-based drug systems have been widely used for therapeutic drug delivery for extended periods of time in a variety of medical illnesses. Such systems require frequent monitoring and replacement of parts because of the possibility of infection, among other untoward effects^8^. Other devices, such as insulin pumps, although somewhat less invasive since they are subcutaneous, also require frequent replacement of both delivery device and medication and have the potential adverse effects of scarring and bleeding^9^. More recently, a subcutaneous implantable device has been proposed for treatment of opioid overdoses^10^. The latter utilizes a burst release design, which has the advantage of on-demand administration of antidote. The device, however, has the disadvantage of housing the medication reservoir subcutaneously, and therefore has an element of invasiveness associated with both implantation and use, including the potential untoward effects of infection, tissue injury, and leakage of drug systemically. It is also quite cumbersome to wear^10^. To overcome these deficiencies, we have designed a wearable automated, burst release, on-demand, compact device for monitoring, reporting, and treating opioid overdoses. Our device uses a simple, integrated power supply to drive a sensor that continuously monitors blood oxygenation via oximetry, and a microcontroller, which responds to critically low oxygen levels, consistent with an overdose, by orchestrating the injection of an opioid antagonist, nalmefene subcutaneously. The microcontroller will also transmit a GPS-trackable 911 alert to inform of the overdose and can be interrogated remotely to provide relevant biometric data. The delivery device is designed to be compact (4 × 3 × 3 cm), and to conveniently attach adhesively to the skin of the upper arm.

## Materials and methods

**Figure 1:**
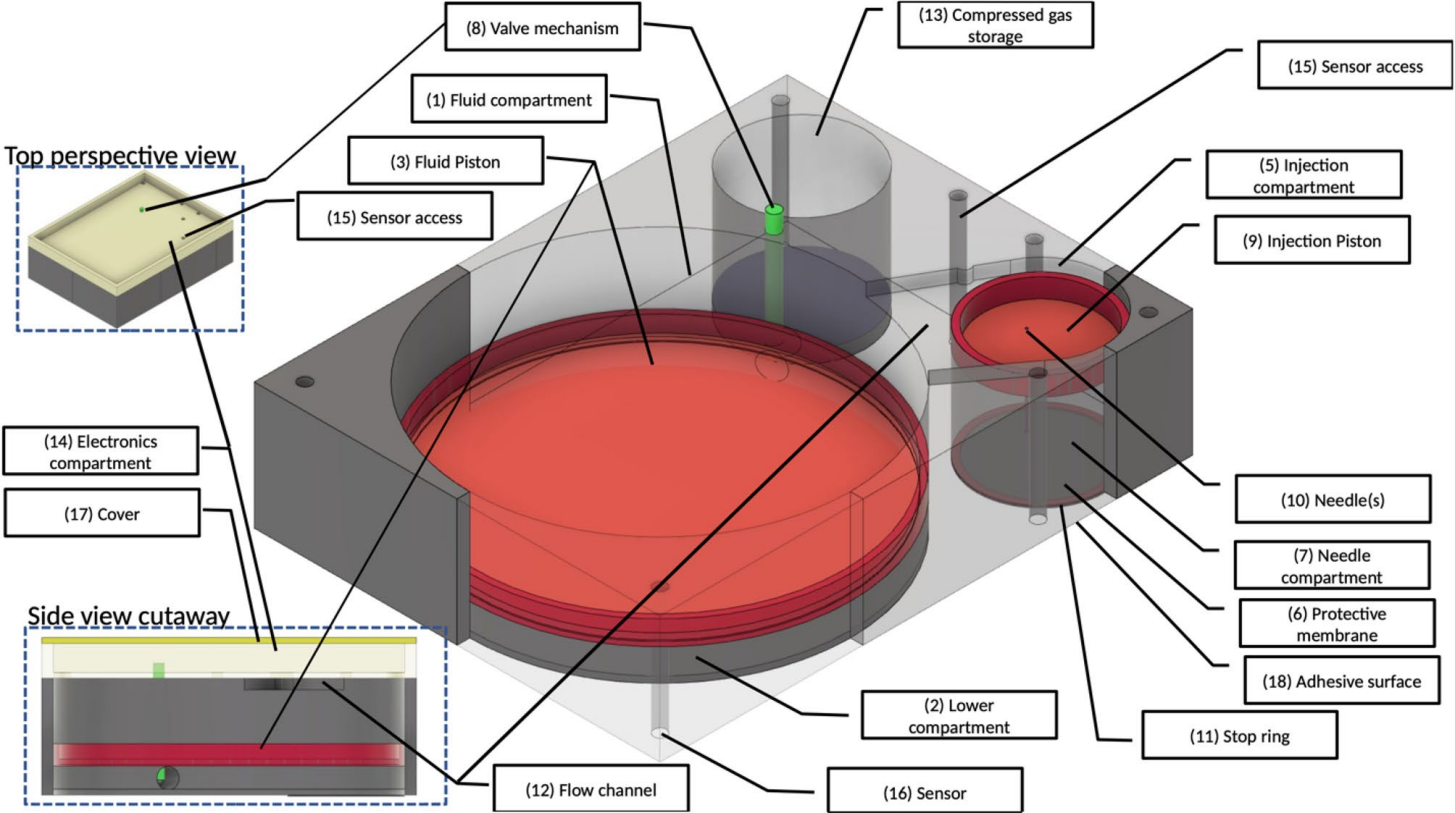
Design overview of the wearable automated self-activated opioid-overdose antidote injection system. Insets show top and side cutaway views. See text for further details.

**Figure 2:**
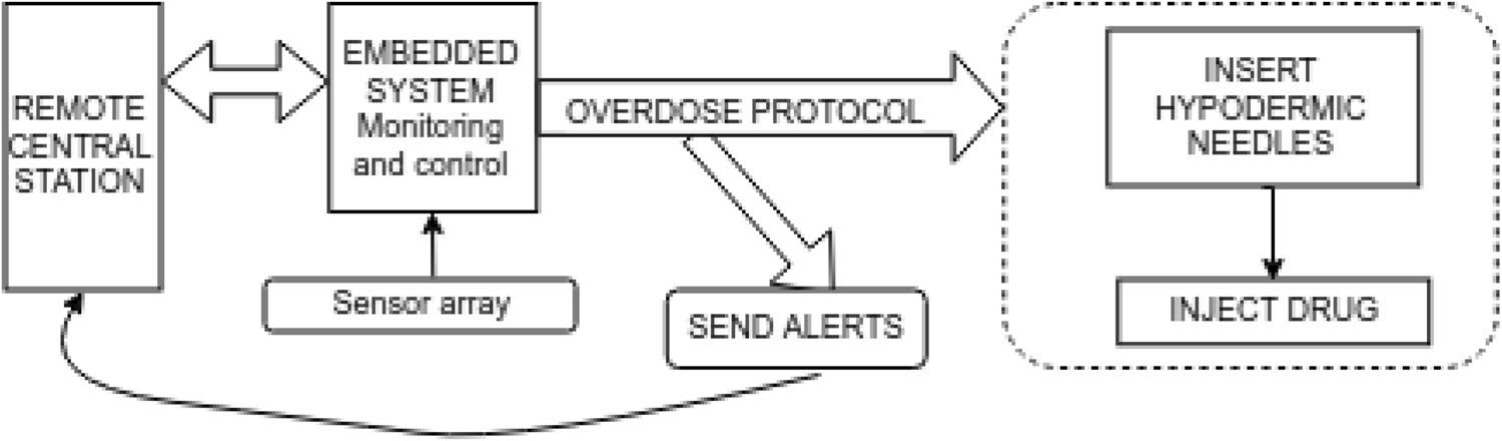
Schematic overview of the opioid-overdose delivery system.

**Figure 3:**
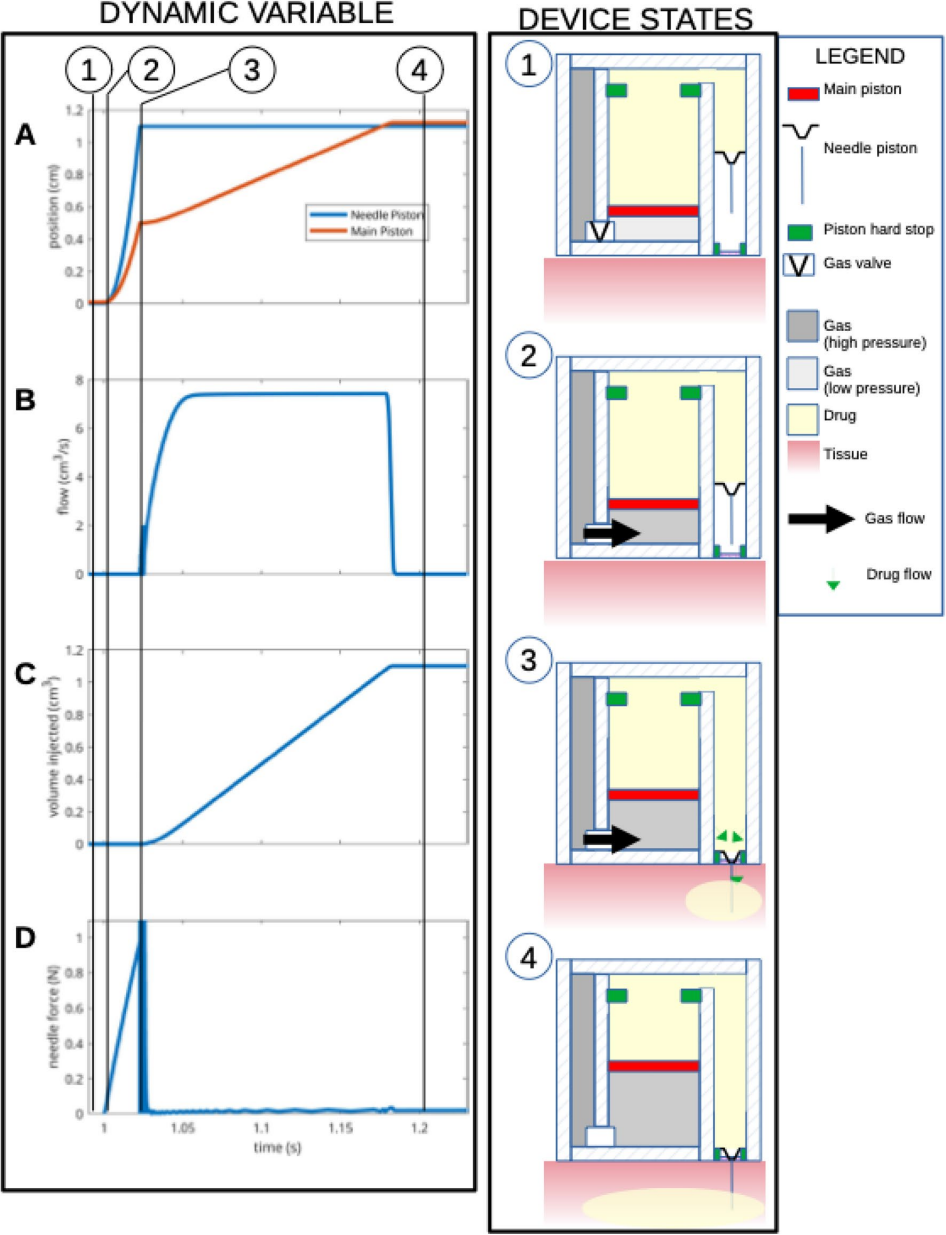
Dynamic model simulation of the mechanical pump system shown for the initial injection cycle. Left panels show time evolution of dynamic variables: (**A**) position of main piston (red trace) and needle piston (blue trace), (**B**) flow of drug through the needle, (**C**) volume of drug injected into the subcutaneous tissue, (**D**) force applied on the needles. Right panels: State of the device at various time points. Balloons 1–4 mark time points which correlate with dynamic states of the device as shown in the left panels 1–4. Shown is a replica of the device mechanics for the initial cycle of engagement following triggering when an SpO2 of less than, or equal to 90% has been sensed by the oximeter. See text for further details.

1. *Control and monitoring*: A microcontroller-based design is used for monitoring and control using a set of sensors. Communication and tracking will be provided by Blue-tooth, WIFI, cellular and GPS tracking modules. A rechargeable battery will provide power to the system.
2. *Mechanical Design*: Needle insertion and drug delivery system.
  The overall mechanical design of the injection device (patent-pending 63/061666) and delivery system (patent pending 63/081579) is presented in Fig. 1. The *Main Fluid Compartment* [Fig. 1] is divided into two parts by the *Fluid piston* [Fig. 1, (3)]. The *Upper compartment* [Fig. 1, (1)] contains fluid (i.e., opioid antidote) and the *Lower compartment* [(Fig. 1, (2)] contains air. The *Lower compartment* (Fig. 1, (2)] is connected via a *Control valve* [Fig. 1, (8)] to the *Compressed gas compartment* [Fig. 1, (13)]. The *Upper main fluid compartment* [Fig. 1, (1)] is connected to the *Injection compartment* [Fig. 1, (5)] via a *Flow channel* [Fig. 1, side view cutaway, (12)]. When triggered, the *Valve* [Fig. 1, (8)] is opened, and gas enters the *Lower fluid compartment* [Fig. 1, (2)] pushing the *Fluid piston* [Fig. 1, (3)] upwards. This drives fluid (i.e., opioid antidote) into the *Injection compartment* [Fig. 1, (5)], which then pushes the *Injection piston* [Fig. 1, (9)] downward. Under the force exerted by the downward movement of the *Injection piston* [Fig. 1, (9)], the *Hypodermic Needle* [Fig. 1, (10)] passes through the *Protective membrane* [Fig. 1, (6)] and penetrates the tissue. Fluid flows only when the *Needle* [Fig. 1, (10)] is fully inserted into the wearer’s subcutaneous tissue. The *Stop ring* [Fig. 1, (11)] then inhibits further movement of the *Injection piston* [Fig. 1, (9)]. The relative movement of pistons and inner assembly is governed by a valve controller. Piston position sensors provide feedback control by which the fluid injection rate and profile are controlled. An algorithm controls the *Gas Valve* [Fig. 1, (8)] to provide a pre-programed drug flow rate. The system is designed to deliver an initial 1 milliliter dose of antidote followed by two additional doses, at 5-minute intervals, in the event of continued oxygen desaturation at either time interval.
3. *Simulation and device parameters*: The mechanical simulation, and device key parameters are given in Table 1. Device maximum dimensions are 4 cm (length), 3 cm (width), 3 cm (height), calculated as follows: (1) Length: Fluid and injection compartments require 3.14 cm. An additional 0.86 cm has been added for walls, resulting in a final length of approximately 4 cm. Width: 2.14 cm width accommodates the fluid compartment (and thus both injection and gas compartments). An additional 0.86 cm has been added for wall thickness, thus generating a final width of 3 cm. Height: 1.1 cm accommodates the cylinder height. An additional 1.9 cm has been added for wall thickness, and the electrical components, resulting in a final height of 3 cm. Compressed gas weight is less than 15 g.
4. Needle: The hypodermic needle to be used to deliver antidote subcutaneously will be 30 gauge, and 8 mm in length (JPN Nanoneedle^11^,).
5. Nalmefene: Nalmefene hydrochloride (cGMP grade) will be purchased from Rusan Pharma Ltd. (Mumbai, India) and prepared as described by others^12^. The sterile, pyrogen-free preparation for subcutaneous administration will be identical to that listed for Revex (nalmefene hydrochloride)^13^ (https:// www.accessdata.fda.gov/drugsatfda_docs/label/2006/020459s006lbl.pdf). The concentration of drug for administration will be 1 mg per milliliter.
6. Oximeter: A reflectance pulse oximeter is integrated into the microcontroller with sensors located as shown in Fig. 1 [item 16]. The microcontroller is programmed to engage the injection device when the percent saturation of hemoglobin with oxygen is less than, or equal to 90%. The biceps location for sensing oxygen saturation has been validated in a recent report^14^.
7. Selection Criteria for Wearing the Device: Our patient population will consist of opioid addicts, particularly those who have recently undergone withdrawal therapy and have elected not to enter a medically assisted arm of treatment, since this group is at high risk for recidivism. Patients entering the protocol will undergo a clinical examination consisting of a history, physical exam and baseline oximetry evaluation. Patients will be excluded from wearing the device if (a) there is a prior, or current history of cardiac or pulmonary disease that might predispose to oxygen desaturation, (b) the physical exam reveals evidence of heart or lung disease that might also predispose to oxygen desaturation, and/or (c) the individual’s percent saturation of hemoglobin with oxygen (SpO2) at rest is less than 93%.
8. Patient follow-up: Individuals who wish to wear the device must submit initially to weekly clinic visits, at which time the device will be checked, components within the device will be replaced if necessary, and the device will be interrogated for compliance. Non-compliance will result in removal from the protocol.

**Table 1:** Simulation and device parameters.

Device module	Description	Value	Units
Fluid cylinder/piston	Piston radius	1.07	cm
	Piston stroke	1.1	cm
	Piston initial displacement	0.01	cm
	Dead volume	1e−5	cm^3^
	Initial pressure	1	atm
Needle cylinder/piston	Piston radius	0.5	cm
	Piston stroke	1.1	cm
	Net hypodermic needle internal diameter	0.6	mm
	Initial pressure	1	atm
Physical chassis parameters	Mass	500	gm
	Spring	11	N/m
	Dashpot	11	N/m
Compressed gas storage	Initial pressure	5	atm
	Radius	0.3	cm
	Height	0.9	cm
Drug fluid properties	Viscosity	0.658	cSt
	Density	992.562	Kg/m^3^

## Results

### Device design overview

This device is designed to be compact and wearable and attaches to the skin using an adhesive surface (Fig. 1, 18). A novel feature of our device is a needle-stow chamber that stores needles in a sterile state and inserts needles into tissue only when drug delivery is needed (see Fig. 1 for details). Figure 2 shows the overall function of the system. An algorithm continuously monitors physiological state of the wearer for an opioid-overdose. A mechanical pump system is activated upon detection of an overdose which inserts the hypodermic needles into tissue, injects the antidote, and simultaneously transmits alerts and data to designated remote systems (e.g., 911 and remote servers). Furthermore, this device can be interrogated remotely for a system health check and the state of the wearer.

The device is composed of two main systems: (1) a microcontroller-based monitoring, reporting, and control system, and (2) a drug delivery system that stores antidote, and stowed hypodermic needle, which, when triggered, inserts the needle into the subcutaneous tissue of the wearer and delivers the antidote (Fig. 1).

A reflectance pulse oximeter^15^ serves as a sensor which continuously feeds physiological data to the microcontroller in the form of percent saturation of hemoglobin with oxygen (SpO2). The microcontroller is programmed to be activated at a SpO2 of less than, or equal to 90%, which corresponds to an approximate partial pressure of oxygen in arterial blood (PaO2) of 60 mm of mercury (normal 80–100 mm of mercury;^16^), and indicates a failure of oxygenation consistent with an opioid overdose in the appropriate setting^7^. Once an opioid overdose is detected, the microcontroller simultaneously transmits a GPS-trackable, 911 alert, and engages the drug delivery system, which then immediately inserts a needle into the subcutaneous tissue of the wearer and delivers 1 ml of antidote. Needle insertion and drug delivery will be complete within ~ 1.2 s of overdose detection (see Fig. 3). The microcontroller is programmed to re-engage the injection device at 5 min intervals × 2 in the event of continued oxygen desaturation at either time interval.

### Device administration and use

The mechanical design of the device has been described in detail above. Briefly, based on a trigger, gas enters the lower part of the *Fluid compartment* [Fig. 1, (2)], which creates pressure, pushing antidote, stored in the upper *Fluid compartment* [Fig. 1 (1)], into the *Injection compartment* [Fig. 1, (5)]. This then drives the *Injection piston* [Fig. 1, (9)] downward thereby inserting the *Hypodermic needle* [Fig. 1, (10)] into the subcutaneous tissue. Once needle placement has been completed, antidote is infused into the tissue. Further movement of the inner assembly is prevented by a *Stop ring* (Fig. 1, 11), and the initial cycle of drug administration ends. *Sensors* [Fig. 1, (16)] embedded in the bottom of the device provide for controlling valve opening, and/or other biometric data to be used by smart control algorithms running on the microcontroller, thus governing the rate of drug injection. The device is equipped with a sufficient supply of gas, and antidote reservoir to accommodate a total of three infusions.

### Mathematical modeling of needle insertion and drug injection

A mathematical model (MATLAB/Simulink, MathWorks Inc.) was formulated to simulate the mechanical dynamics of the design. Figure 3 illustrates the first injection cycle, which is approximately1.2 s in duration. Initially the device is in a ready state (panel A-D, balloon 1, t < 1 s). At t = 1 s, the device is triggered, causing compressed gas to flow in the lower chamber (balloon 2). This causes the main piston to move (panel A red trace, & balloon 2). We have assumed the fluid, nalmefene, is incompressible, thus force is transferred to the injection piston immediately causing it to move downwards along with the needle, (panel A blue trace, & balloon 2). At this stage no fluid is flowing through the needles as there is no substantial pressure increase in the fluid chambers due to the moving pistons. As the hypodermic needle continues its descent, it penetrates the sterile protective membrane and enters the tissue. Once the needle piston has traveled its maximum distance (panel A blue trace), the hypodermic needle is fully inserted (~ 8 mm) into the subcutaneous tissue (balloon 3). At this stage maximum needle force is achieved (> 1 N, panel D). As the main piston continues to move upwards (red trace, panel A), flow of fluid in the subcutaneous tissue starts (panel B & C). The volume injected is monitored by the control algorithm via the main piston position. Once 1 ml volume of the antidote has been infused, flow of gas ceases. The initial cycle is completed within ~ 1.2 s, by the end of which ~ 1 ml of drug has been injected into the tissue (balloon 4). After 5 min, if the SpO2 continues to remain at less than or equal to 90%, the microcontroller will again engage the injection device to infuse an additional 1 ml of nalmefene into the subcutaneous tissue. A third engagement of the injection device will be repeated 5 min after the second infusion if the SpO2 continues to be less than or equal to 90%.

## Discussion

Deaths due to opioid overdoses continue to increase at an alarming rate^17^ and have been described by the CDC as a worsening and expanding epidemic^18^. Beginning in 2008, unintentional overdose deaths among adults between the ages of 24 and 65 have exceeded motor vehicle crash deaths, and suicides as a leading cause of injury death in the United States^6^. More recently, a sharp rise in the number of opioid-involved fatalities occurred in during the COVID 19 pandemic^19^, and a call for enhanced prevention and response measures has been made^20^. A major impediment to saving lives lost to opioid overdoses is the lack of timely administration of antidote, which, in part is due to the fact that overdose cases may occur absent antidote^6^, or may be unwitnessed, and the opioid itself may render the victim incapable of self-administration of antidote^7^. To directly address this problem, we have designed a novel, non-invasive, conveniently worn device that provides a continuous monitoring, and reporting system, and a self-activating, safe, antidote delivery system that administers the antidote automatically when needed.

We wanted to create a wearable device that would be free of the potential complications of infection inherent in intravenous and intraarterial delivery systems, which necessitates frequent replacement of needles and/or cannula^8^. Unlike in-dwelling subcutaneous devices^10^, the ideal device should not have the potential untoward effect of leakage, require re-entry into subcutaneous tissue on replacement, or subject the host’s tissue to prolonged exposure to a foreign body. Moreover, the ideal delivery system should also be minimally invasive, that is, antidote should be administered only when required. Furthermore, antidote delivery should occur automatically; administration should be independent of cognition and physicality (which, as stated earlier, are frequently impaired in the setting of an opioid overdose), and the timing of injection should be determined by evidence-based, physiological parameters. Finally, the device should be compact and unobtrusive, so it can be conveniently worn externally.

The system we have designed satisfies all of the above criteria for an ideal device to treat an opioid overdose. Our delivery device is non-invasive, and adhesively attaches to the skin. It is unobtrusive, being 4 × 3 × 3 cm in size, and can be conveniently worn on the upper arm. Our monitor is a reflectance pulse oximeter which is integrated into, and drives the microcontroller to inject antidote subcutaneously in response to a physiologically sound, medically relevant indicator i.e., dangerously low oxygenation of blood, or hypoxia.

The oximeter, which measures SpO2, has been validated as a means of detecting hypoxia both experimentally^21^, and clinically^22^. When comparing pulse oximetry readings with direct measurements of percent saturation of arterial blood with oxygen (SaO2), the standard deviation of difference (SpO2-SaO2) was 4.7% over a SaO2 range of 17% to 100%^23^. Thus, pulse oximeter measured oxygen saturation is a well-accepted, non-invasive indication of arterial oxygen saturation^15,24^, and has been considered the fifth vital sign in clinical assessment^25^. To strengthen the specificity of our alarm, without significantly compromising sensitivity, we have included criteria for individuals to be permitted to wear the device. Thus, all subjects will be required to give a history, take a physical exam, and undergo baseline SpO2 testing to rule out intrinsic, pre-existing, or current cardiopulmonary disease that might predispose to oxygen desaturation, or affect the capacity to maintain an SpO2 of 93%, the generally accepted lower limit of normal^26^.

Although diurnal fluctuations in our SpO2 and corresponding PaO2 occur periodically^27^, there is an accepted normal range of SpO2 of 93 to 97%, equivalent to an approximate range of PaO2 of 80 and 100 mm of mercury, respectively. An SpO2 of less than 93% is abnormal and, if consistently exhibited, requires medical attention. An SpO2 value that is less than, or equal to 90% is pathological and indicates that the individual is hypoxic, and minimally, in need of supplemental oxygen^28,29^.

In deciding on an SpO2 that would act as a threshold to engage the injection device in our targeted population, we asked what level of oxygen desaturation would permit the greatest chance of both cognitive and physical recovery from an overdose while compromising specificity least. Since optimal SaO2 occurs at a PaO2 of 80 to 100 mm of mercury, and cell viability is highly sensitive to oxygen deprivation, we reasoned that the greatest chance of preservation of tissues in the setting of oxygen desaturation would occur at the onset of hypoxia, defined here as the SpO2 at which administration of exogenous oxygen is required in order to optimally prevent cell death. Evidence based guidelines have established the mean SpO2 at which oxygen should be administered (i.e., the onset of hypoxia) to be 90%^28,29^. This represents the SpO2 threshold below which tissue viability is at risk. We therefore chose to be conservative and initiate antidote treatment at the threshold of the hypoxic event, rather than waiting for the appearance of severe hypoxemia, which may ultimately lead to profound compromise of cognitive and physical function, notwithstanding the possible risk of increasing our rate of false positive reporting. In our selected, targeted population, a sudden, precipitous decline of the SpO2 from the normal range (93–97%) to less than or equal to 90%, corresponding to a PaO2 of less than or equal to 60 mm of mercury, should strongly suggest an opioid overdose^7^.

Following detection of oxygen desaturation the microcontroller simultaneously initiates a GPS trackable, 911 alert, and engagement of the injection device, resulting sequentially in the downward movement of the hypodermic needle, penetration of the subcutaneous tissue of the victim, and administration of one milliliter of nalmefene within 1.2 s of the trigger (Fig. 1). This process is repeated at 5 min intervals × 2 if the SpO2 remains less than, or equal to 90% at either time interval. Since our device transmits a remote alert, we assume that first responders will arrive on scene to provide treatment by the time a 4th dose of nalmefene would be administered (i.e.,15 min after signal activation). Should the victim still manifest signs of an overdose at that time, first responders will have the option of administering a standard dose of 2 mg of naloxone, as has been used as a rescue antidote by others in a single nalmefene refractory case^30^.

Our choice of nalmefene as an antidote for opioid overdoses over conventionally used naloxone was partially based on the fact that the death rates of opioid overdoses have been increasingly linked to fentanyl and its analogs. In fact, these synthetic opioids, which are highly potent and have a relatively long half-life, accounted for nearly 73% of all opioid deaths in 2019 (cdc.gov/drugoverdose/data/synthetic/index.html). Given this, the pharmacological properties of nalmefene suggest that it may be more effective as a rescue agent than naloxone for treating overdoses caused by the newer high affinity, long acting, synthetic opioids^31–33^. Clinically, nalmefene’s plasma half-life is 8 h, greater than 2.5-fold longer than naloxone, and comparable to that of the synthetic opioid, fentanyl^34^. Nalmefene also has a greater affinity than naloxone for mu opioid receptors^5,12,35,36^, and an experimental study in rats has shown that nalmefene is more potent than naloxone in reversing carfentanyl induced respiratory depression^37^.

Nalmefene was initially shown to be clinically effective as a rescue agent in an open label trial in patients with suspected overdoses^38^. The efficacy of nalmefene relative to naloxone, was subsequently compared in a double-blind, randomized trial^30^. No statistical differences in efficacy or withdrawal outcomes were seen between groups that received nalmefene 1 mg, or naloxone 2 mg, and no significant overall time-treatment interactions occurred, among opioid-positive cases.

The report of Kaplan et al^30^ showed no significant differences in outcome between nalmefene 1 mg compared with a dose of 2 mg when treating an opioid overdose, however, a greater incidence of side effects has been reported with doses of greater than 1 mg of nalmefene^30,33^, and/or when four doses of either nalmefene 1–2 mg or naloxone 2 mg were employed^30^. After balancing safety with the current trend towards synthetic, high potency opioid abuse, we elected to use an initial dose of 1 mg of nalmefene, and a maximum of 3 doses of nalmefene in our device to effect opioid overdose reversal.

Our device is GPS- trackable, capable of transmitting a 911 alert upon activation, and can be interrogated remotely, which permits real-time access to physiological data, and information on whether or not the device is actually being used. It is primarily intended for those at high risk for an opioid overdose^6,39,40^. These include opioid addicts, particularly those individuals who have recently undergone opioid drug withdrawal and have elected not to enter a medical assisted arm of therapy, since this population is at high risk for recidivism. Individuals who are also at significant risk for unintentionally overdosing with opioids, such as patients chronically taking opioids for medically supervised pain management^41^, as well as recreational drug users may benefit from using the device.

There are inherent weaknesses associated with our automated delivery system and protocol design. Compliance in wearing the device may pose a problem, and there are additional costs associated with screening and patient follow-up. However, these costs pale in the face of the overall economic burden to society of the opioid use disorder, and associated opioid related deaths^42^. Therefore, if clinical studies demonstrate that in opioid overdose victims our device can effectively prevent both visits to the emergency department, and hospitalizations, and save lives as well, the monetary savings will become self-evident, and justify its use.

## Conclusions

Many lives are lost due to unintentional opioid overdoses. Unfortunately, opioid-overdoses may occur in the absence of antidote, or may be unwitnessed, and the rapid onset of cognitive impairment and unconsciousness, which frequently accompany an overdose may render self-administration of an antidote impossible. Currently no solutions are available that are practical for extended, safe use, which provide monitoring and automatic administration of antidote. In this investigation we present the design of a wearable, on-demand system that comprises a safe, compact, non-invasive device which can monitor, and effectively deliver an antidote without human intervention, and report the opioid overdose event. Furthermore, this device permits a centralized, remotely accessible system for effective institutional, large-scale intervention. Most importantly, this device has the potential for saving lives that are currently being lost to an alarmingly increasing epidemic.
